# A comparison of role‐related physical fitness between British Army trainees and trained soldiers

**DOI:** 10.1002/ejsc.12227

**Published:** 2024-12-02

**Authors:** Carla Rue, Sarah Needham‐Beck, Tessa Maroni, Andrew Siddall, Kimberly Ashdown, Ben Lee, Faye Walker, Joshua Osofa, Julianne Doherty, Christopher Vine, Sophie Wardle, Julie Greeves, Paul Saunders, Anne Fieldhouse, Sam Blacker, Stephen Myers

**Affiliations:** ^1^ Occupational Performance Research Group University of Chichester Chichester UK; ^2^ Faculty of Health and Life Sciences Coventry University Coventry UK; ^3^ Department of Army Health and Performance Research UK Ministry of Defence Andover UK; ^4^ Department of Targeted Intervention University College London London UK; ^5^ Norwich Medical School University of East Anglia Norwich UK

**Keywords:** endurance, military fitness, occupational performance, strength

## Abstract

British Army basic training (BT) and initial trade training (ITT) enable personnel to develop role‐related physical capability to perform in‐service job‐roles. The study aimed to compare physical performance of trainees (completing ITT) and trained soldiers, on a series of gym‐based fitness tests and representative military tasks. A total of 316 British Army personnel [68 trainees (63 men: 22 ± 3 years, 71.6 ± 8.4 kg and 1.74 ± 0.07 m) and 248 trained soldiers (225 men: 27 ± 6 years, 78.7 ± 12.7 kg and 1.76 ± 0.08 m)] completed two sessions. Session 1; body mass, stature, age and gym‐based tests (2 km run, broad jump, seated medicine ball throw, hex bar deadlift, 100 m shuttle sprints, pull‐ups and mid‐thigh pull). Session 2; representative military tasks (loaded carriage [stage 1, 4 km, 35–40 kg and 4.8 km h^−1^ fixed pace and stage 2, 2 km, 20–25 kg and individual best‐effort speed], tactical movement, casualty drag, stretcher carry, vertical lift, repeated carry and incremental lift). Independent sample *t*‐tests were employed to examine group differences. Compared to trainees, trained soldiers were older (*p* < 0.001), heavier (*p* < 0.001) and scored higher on broad jump (*p* = 0.024), medicine ball throw (*p* = 0.007) and mid‐thigh pull (*p* = 0.048), but were slower on 2 km run (*p* = 0.047), loaded carriage (*p* < 0.019), tactical movement (*p* < 0.001) and casualty drag (*p* < 0.001). Overall, trainees achieve higher scores on aerobic/anaerobic tests, whereas trained soldiers outperform trainees in strength/power‐based tests. Although a cross‐sectional comparison does not provide strong evidence, the results may indicate that cardiovascular fitness is developed during BT, whereas muscle strength/power develops post BT/ITT. These findings would need confirming by a longitudinal study and could inform the development/management of role‐related fitness during BT, ITT and through career.

## Highlights

- The Role Fitness Test (Soldier) and Soldier Conditioning Review performance of British Army trainees and trained soldiers have not previously been examined. Establishing whether differences in performance between the populations exist will help inform the understanding of the physical development of soldiers after ITT/through career.
- This cross‐sectional study indicates that cardiovascular fitness is developed during BT, whereas muscle strength/power develops post BT/ITT; however, these findings would need confirming by a longitudinal study.
- Trainees should be provided with sufficient opportunities to practice strength and power‐based tests throughout ITT to enable them to meet physical employment standards.

## 1 INTRODUCTION

British Army soldiers complete a period of basic training (BT) followed by initial trade training (ITT) to develop the physical fitness and technical capabilities required to perform their role in service. Soldiers are required to undertake role‐related tasks including loaded carriage, casualty evacuations, tactical movements and material manual handling. Collectively, these role‐related tasks require a combination of key components of fitness: cardiorespiratory (or aerobic) endurance, anaerobic endurance, muscle strength, muscle endurance and mobility (Blacker et al., 2009). The development of these components of fitness are an important requirement for both BT and ITT, before trained soldiers complete ongoing physical training to further develop and maintain physical readiness through career.

In 2021, the British Army introduced new gender‐ and age‐free physical employment standards for Non‐Ground Close Combat (Non‐GCC) personnel. These physical employment standards, termed as the Role Fitness Test (RFT) by the British Army, are used at the point of entry [RFT (Entry); RFT(E)] during basic training [RFT (Basic Training); RFT (BT)] and annually in‐service [RFT (Soldier); RFT(S)] to assess the physical ability of trainees and soldiers to undertake their role. The development of these physical employment standards were informed using a job task analysis, which quantified the physically demanding job tasks conducted by British Army personnel (Blacker et al., 2020, 2021). The new physical employment standards were designed to assess the components of fitness required to perform the Non‐GCC job roles. The RFT(E) and RFT (BT) both consist of a 2 km run, seated medicine ball throw and a mid‐thigh pull. The RFT(S) consists of role‐related representative military tasks including a loaded carriage, tactical movement, casualty drag, stretcher carry, vertical lift, repeated carry and an incremental lift test. During the implementation of the RFT, the British Army also introduced the Soldier Conditioning Review, which is used to review and monitor trained soldiers' physical capability through a series of gym‐based fitness tests. The Soldier Conditioning Review tests include the broad jump, seated medicine ball throw, hex bar deadlift, 100 m shuttle sprints, pull‐ups and 2 km run.

Previously, Canino et al. (2019) reported differences in physical performance between unmatched trainees and active duty soldiers on the United States Army Physical Fitness Test (push‐ups, sit‐ups and 2‐mile run) and three role‐related tests (sandbag carry, casualty drag and move under direct fire). Using age, height and mass as covariates, the results demonstrated that active‐duty soldiers were able to complete a higher number of push‐ups and sit‐ups as well as perform a faster sandbag carry and casualty drag than trainees. However, trainees were faster on the best‐effort 2‐mile run. Although differences were apparent, most trainees successfully attained the minimal acceptable performance standard in these assessments demonstrating the efficacy of their initial entry training (Canino et al., 2019). In support of these findings, Dada et al. (2017) also reported that male and female operational soldiers had greater muscle and cardiorespiratory endurance than basic combat training soldiers, when assessed on the Army physical readiness test (Dada et al., 2017).

The physical performance of British Army trainees and trained soldiers has not previously been compared. Establishing whether differences in performance exist between these two groups will help inform the understanding of the physical development of soldiers after ITT and through career. This information could help inform future strategies to optimise physical development of personnel. Therefore, the aim of this study was to compare physical performance on the gym‐based tests and role‐related representative military tasks between British Army Non‐GCC trainees and trained soldiers.

## 2 MATERIALS AND METHODS

Using a cross‐sectional study design, 316 British Army personnel completed the study (Table 1). Data were collected over several periods, spanning 8 weeks from February to April 2020, in trainee (i.e. Army Medical Services, Royal Artillery, Royal Electrical and Mechanical Engineers and Royal Engineers) and trained (i.e. Army Air Corps, Adjutant General's Corps, Army Medical Services, Intelligence Corps, Royal Artillery, Royal Electrical and Mechanical Engineers, Royal Engineers, Royal Logistic Corps, Royal Military Police and Royal Corps of Signals) military cohorts. All participants received a comprehensive verbal and written brief before providing written informed consent. Participants were reminded of the right to withdraw at any point and were advised of withdrawal procedures used by the overseeing researchers or physical training instructors if the techniques performed were deemed unsafe at any stage. The study was given favourable opinion by the Ministry of Defence Research Ethics Committee (Application no: 993/MODREC/19).

**TABLE 1:** Participant characteristics and rank (mean ± SD).

Group	Trainees			Trained soldiers		
	All	Men	Women	All	Men	Women
Sample size (n)	68	63	5	248	225	23
Age (y)	22 ± 3	22 ± 3	20 ± 2	27 ± 6	27 ± 6	24 ± 6
Body mass (kg)	71.6 ± 8.4	71.8 ± 8.6	70.0 ± 6.2	78.7 ± 12.7	79.9 ± 12.5	66.5 ± 6.4
Stature (m)	1.74 ± 0.07	1.75 ± 0.07	1.66 ± 0.08	1.76 ± 0.08	1.77 ± 0.07	1.67 ± 0.07
Body mass index (kgm^2^)	23.6 ± 2.3	23.4 ± 2.3	25.3 ± 1.6	25.3 ± 3.0	25.5 ± 3.0	24.0 ± 2.2
Private/Equivalent	68	63	5	109	99	10
Lance corporal/equivalent	0	0	0	75	68	7
Corporal/Equivalent	0	0	0	28	25	3
Senior non‐commissioned officer/equivalent	0	0	0	36	33	3

Stature (m) and body mass (kg) were measured and recorded prior to the gym‐based tests for all participants, from which body mass index (BMI: kg·m^2^) was calculated. The physical capabilities of participants were assessed using seven gym‐based tests (2 km run, seated medicine ball throw, mid‐thigh pull, broad jump, hex bar deadlift, 100 m shuttle sprints and pull‐ups) and seven representative military tasks (loaded carriage, tactical movement, casualty drag, stretcher carry, vertical lift, repeated carry and incremental lift) over two sessions. For trainees, gym‐based tests were completed in week 1 and representative military tasks in week six of ITT. The 6‐week time point was agreed between researchers and military stakeholders to be the most appropriate place to conduct the representative military tasks, due to the highly varied duration and content of ITT. For trained soldiers, gym‐based tests and representative military tasks were separated by 48 h. Participants were familiarised with all the tests, with a physical training instructor* demonstrating each one using proper technique. All tests were overseen by physical training instructors and performed to best‐effort.

### 2.1 Gym‐based tests

Participants completed a warm‐up led by physical training instructors including an 800 m run at 8 km h^−1^ determined via a wrist‐worn GPS device followed by dynamic stretches. The 2 km run was completed first followed by the remaining tests in a randomised order, with a minimum of 5 minutes' rest between all tests. At each training establishment, tests were conducted in groups of 10–15, except for the 2 km run in which the group size ranged from 10 to 50. Where multiple efforts for a test were required (e.g. broad jump), a minimum of 30 s recovery separated each effort. All participants wore standard‐issue sports shorts, t‐shirt and running shoes.

The 2 km run was conducted along a pre‐measured flat tarmac outdoor route unique to each test location. Time to complete the 2 km run was recorded to the nearest second.

Participants completed two best‐effort medicine ball throws. Participants were required to sit with their back and shoulders against a wall with legs fully extended whilst holding a 4 kg medicine ball (Loumet medicine ball, Per‐form Better Ltd, Southam, UK) at chest height, with elbows facing downwards. On command, participants forcefully pushed the medicine ball upwards and outwards to achieve a maximum distance throw. Throw distance of both best‐effort attempts was measured from the wall behind the participant to the landing point of the ball and recorded to the nearest 0.05 m. The furthest recorded distance was used for analysis.

To perform the mid‐thigh pull test, participants stood on a weighing scale (MW Model scale with Cardinal 190 indicator, AWM Ltd, UK) housed within a specially manufactured rig, which fixes a bar at an adjustable height (AP‐IPAT01, Absolute Performance Limited, UK). With participants' feet shoulder width apart and knees slightly flexed, the bar was moved to approximately mid‐thigh level to acquire a hip angle between 140° and 150° and a knee angle between 120° and 135° (Beckham et al., 2018). Participants took hold of the bar with an overhand grip and straight arms, then following instruction to “take up the slack” on the bar (lifting the bar to meet upward resistance) and a brief pause; participants exerted a maximal isometric force by gradually pulling upwards as hard as possible and maintained the pull for ∼5 s. Peak absolute force (kg) was recorded during two best‐effort attempts. The highest peak force generated was used for analysis.

Participants completed two best‐effort standing broad jumps. Participants were required to stand with their feet behind a line marked on a flat surface. On command, using a two‐footed take off, participants jumped as far forward as possible landing on both feet to achieve a maximum distance jump. Jump distance of both best‐effort attempts was measured from the line to the heel of the shod foot closest to the line and recorded to the nearest 0.05 m. The furthest distance jumped was used for analysis.

To perform the hex bar deadlift, participants were required to lift a hex bar with an overhand grip lifting from the lower back and shoulder girdle until their legs are straight at the knees with the bar at mid‐thigh height. Following a warm‐up of 10 repetitions at 40 kg, participants self‐selected (in 10 kg increments) the mass on the bar and were asked to complete as many repetitions as possible (up to a maximum of 10). If participants achieved the 10 repetitions, they were permitted to increase the mass lifted (a maximum of 20 kg) and repeat the procedure. If unable to complete 10 repetitions or technique, it was deemed poor; the total number of repetitions was recorded and the test ended. The maximum number of repetitions at a given lift mass was used to estimate one repetition maximum (1RM) using supplementary Table A.1 (Army fitness tests, 2022).

The 100 m shuttle sprints were completed following a warm‐up of 2 × 20 m shuttles at 50%–75% effort. Once complete, participants were required to adopt a prone position facing the starting direction of the first sprint with shoulders in line with the start line on a flat surface. On command, participants assumed an upright position and sprinted 20 m as quickly as possible before touching their foot on a line and turning around to complete another 20 m sprint. This action was repeated until five 20 m sprints were completed. Total time taken to cover the 100 m was recorded to the nearest second.

To perform the pull‐ups, on command, using an overhand grip, participants were required to pull their body from an extended arm position (such that their head and chin is above the bar) and lower their body to an extended arm position in time with instruction from a physical training instructor, for example, “UP” and DOWN”. Participants continued until failure or were unable to keep in time or lost form. The total number of pull‐ups completed was recorded.

### 2.2 Role‐related representative military tasks

Before the completion of the representative military tasks, participants completed a thorough warm‐up led by physical training instructors. All participants completed the tests in the order presented within Supplementary Table A.2, with a minimum of 5 minutes' rest between all tests. Tests were conducted in groups of 10–15 participants except for the loaded carriage in which the group size ranged from 10 to 50. All participants wore fatigues and boots, alongside a combination of webbing, weapon, Bergen/daysack, body armour and helmet as specified with corresponding masses in Supplementary Table A.2 for each role‐related test.

The loaded carriage was divided into two stages and conducted on a flat pre‐measured outdoor route unique to each test location. During stage 1, participants were required to cover 4 km in 50 min (paced by physical training instructors at 4.8 km h^−1^ determined via a wrist‐worn GPS device), as a squad. During stage 2, participants were required to cover 2 km to an individual best effort. Since stage 1 was paced, completion time for stage 2 of the loaded carriage was recorded to the nearest second.

Participants started the tactical movement in a prone position on the ground. On command, they assumed an upright position and completed a series of 7.5 m bounds totalling 90 m. Each bound is completed in 8 s, with an inter‐bound rest of 8 s (in time to an audio track). On completion of the 90 m, participants completed an individual best effort 7.5 m leopard crawl and 7.5 m sprint. Completion time for the 7.5 m leopard crawl and 7.5 m sprint (combined) was recorded to the nearest second.

For the casualty drag, participants were required to drag a 110 kg bag in a backwards direction over 20 m as quickly as possible (Vine et al., 2023). Completion time was recorded to the nearest second.

To perform the stretcher carry, participants were required to carry two 22 kg water cans over 240 m (8 × 30 m) as fast as possible. Placing the water cans down and resting when necessary was permitted. Completion time was recorded to the nearest second.

To perform the vertical lift, participants stood on two boxes placed 0.49 m apart and adopted a deep squat position grasping a rope (at the level of their feet) with weight plates attached. On command, participants stood up, with arms and legs extended, lifting the weight plates, before holding for 3 s and lowering under control. Starting lift mass was 40 kg and increased in 10 kg increments with each successful attempt to a maximum of 110 kg. A minimum recovery of 30 s was provided between attempts. The maximum mass successfully lifted, held and lowered under control was recorded in kilograms.

For the repeated carry, participants were required to pick up 2 × 22 kg water cans from the floor and carry 30 m (out 15 m and back), before placing down on the floor and walking back 15 m without load, they then picked up 1 × 20 kg Powerbag™ from the floor and carried it 30 m (out 15 m and back), before placing down on the floor and completing 15 m unloaded. This was repeated as many times as possible in 10 min. The total number of shuttles (out and back) completed was recorded.

For the incremental lift, participants were required to perform a maximum lift to three different heights. Firstly, participants lifted a Powerbag™ from the floor and placed it on a 1 m platform. Then, they lifted from the 1 m platform to a “front rack” shoulder position, before pausing and continuing to lift the Powerbag™ overhead, finishing with arms straight. This was repeated with increasing masses, starting with a 15 kg Powerbag™, and increasing in 5 kg increments, until failure or up to a maximum lift of 50 kg. Recovery was provided between attempts. The maximum mass lifted safely to (a) 1 m platform, (b) shoulder, and (c) overhead was recorded in kilograms.

### 2.3 Population‐wide Soldier Conditioning Review

Anonymised performance data for 44,469 trained soldiers on the six Soldier Conditioning Review tests (broad jump, seated medicine ball throw, hex bar deadlift, 100 m shuttle sprints, pull‐ups and 2 km run) were summarised from records held on the British Army Fitness Information Software System (FISS) database. These data were used to describe whether physical performance of trained soldiers on the broad jump, seated medicine ball throw, hex bar deadlift, 100 m shuttle sprints, pull‐ups and 2 km run in the present study were representative of personnel in the wider British Army.

Statistical analysis was conducted using JASP (v0.16.3, University of Amsterdam, Netherlands), with data presented as mean ± standard deviation unless stated otherwise. Data normality was assessed using the Shapiro–Wilk test. To examine group differences in demographics, gym‐based and role‐related representative military tasks, independent samples student *t*‐tests (*t*) and Mann–Whitney U tests (*Z*) were applied to parametric and non‐parametric data, respectively. Significance level was set at *p* < 0.05.

## 3 RESULTS

Group characteristics are shown in Table 1. Compared with trainees, trained soldiers were ≈5 years older (*p* < 0.001), 19% heavier (*p* < 0.001) and had 7% greater body mass index (*p* < 0.001) but were not different in stature (*p* = 0.130). Performances on the gym‐based and role‐related representative military tasks are summarised in Table 2. Trained soldiers scored higher than trainees on the broad jump (*p* = 0.035), seated medicine ball throw (*p* = 0.007) and mid‐thigh pull (*p* = 0.048), whereas trainees performed better on the 2 km run (*p* = 0.034). There were no other statistically significant differences in performance on the gym‐based tests. For the representative military tasks, trainees performed better on stage 2 of the loaded carriage (*p* = 0.007), tactical movement (*p* < 0.001) and casualty drag (*p* < 0.001). No differences were found in stretcher carry, vertical lift, repeated carry or incremental lift performance between the trainees and trained soldiers.

**TABLE 2:** Performance scores for gym‐based and role‐related representative military tasks for trainees and trained soldiers (mean ± SD).

Test	Trainees	Trained soldiers	Test statistic	*p*
Gym‐based tests				
2‐Km run (min:s)	08:17 ± 01:03	08:34 ± 00:57	*Z* = 9850.5	0.034
Broad jump (m)	1.85 ± 0.25	1.93 ± 0.25	*Z* = 9836.5	0.035
Seated medicine ball throw (m)	4.21 ± 0.65	4.47 ± 0.72	*t* = 2.700	0.007
Hex bar deadlift 1RM (kg)	120 ± 21	121 ± 24	*Z* = 8648.0	0.746
100 m Shuttle sprints (s)	23.07 ± 1.37	22.83 ± 1.70	*Z* = 7295.0	0.089
Pull‐ups (n)	7 ± 4	6 ± 4	*Z* = 7325.5	0.097
Mid‐thigh pull (kg)	148.51 ± 27.51	156.06 ± 30.14	*Z* = 9751.5	0.048
Representative military tasks				
Loaded carriage (min:s)	12:52 ± 02:22	13:36 ± 01:38	*Z* = 10,231.0	0.007
Tactical movement (s)	11.95 ± 3.43	13.58 ± 3.43	*Z* = 11,335.0	<0.001
Casualty drag (s)	20.63 ± 10.49	28.62 ± 13.65	*Z* = 12,806.0	<0.001
Stretcher carry (min:s)	02:12 ± 00:45	02:14 ± 00:39	*Z* = 9491.0	0.113
Vertical lift (kg)	97 ± 15	96 ± 15	*Z* = 8314.5	0.855
Repeated carry (no. of shuttles)	34 ± 7	35 ± 5	*t* = 0.790	0.430
Incremental lift (1 m) (kg)	46 ± 5	46 ± 6	*Z* = 8551.0	0.840
Incremental lift (shoulder) (kg)	38 ± 7	39 ± 7	*Z* = 9128.5	0.286
Incremental lift (overhead) (kg)	33 ± 8	34 ± 8	*Z* = 9424.5	0.129

**FIGURE 1:**
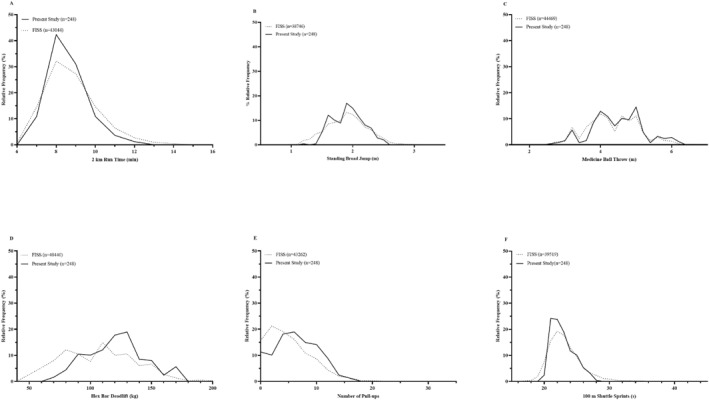
Comparison of distribution of performance scores for the trained soldier cohort from the study cohort versus wider Army population from the Fitness Information Software System (FISS) for each of the six Soldier Conditioning Review tests (A) 2 km run (B) standing broad jump, (C) seated medicine ball throw, (D) hex bar deadlift, (E) pull‐ups and (F) 100 m shuttle sprints. The curves show the distribution of the individual tests from the trained soldiers (solid) and FISS data (dashed).

The distribution of Soldier Conditioning Review performance data of trained soldiers in the study cohort compared to records held on the FISS database shown in Figure 1 indicate that participants' physical performance in the present study were representative of the wider British Army.

## 4 DISCUSSION

This study compared the physical performance of non‐GCC trainees and trained soldiers across a series of gym‐based and role‐related fitness tests. Results showed that trained soldiers compared with trainees were older, heavier and scored higher on gym‐based strength‐ and power‐tasks such as the broad jump, seated medicine ball throw and mid‐thigh pull. Conversely, trainees performed better on the 2 km run, loaded carriage, tactical movement and casualty drag than trained soldiers. No further differences in gym or representative military tasks between trainees and trained soldiers were observed. Data collected from the study cohort of trained soldiers are representative of performance from the wider Army population on the Soldier Conditioning Review.

The current study demonstrated that trainees outperformed trained soldiers in tasks where aerobic endurance, anaerobic endurance and mobility were the principal component of fitness underpinning performance (i.e. 2 km run, loaded carriage, tactical movement and casualty drag). These results are likely due to the greater emphasis placed on cardiorespiratory fitness during BT, which trainees had completed more recently than trained soldiers. Basic training has been well documented to significantly improve aerobic endurance (Dyrstad et al., 2006; Harwood et al., 1999; Rosendal et al., 2003; Santtila et al., 2008; Varley‐Campbell et al., 2018; Williams, 2005), supporting and helping to contextualise this finding. Interestingly, although studies have demonstrated that aerobic fitness within BT regresses to a whole cohort mean (Burley et al., 2018; Rue et al., 2023), these data suggest that the level of performance is still higher than that of individuals within non‐GCC roles. Plausibly the difference between trainees and trained soldiers could be explained by differences in physical training programmes between the two cohorts; trainees complete structured training, whereas trained soldiers complete more self‐directed training, which is subject to more disruption (e.g. operational duties).

Upper and lower body muscle power determined by the seated medicine ball throw and broad jump was shown to be greater in trained soldiers. Since the seated medicine ball throw has been associated with skeletal muscle mass and correlated with lower body power (Aandstad, 2020), this result alongside greater broad jump scores indicates that muscular power develops during a service career following BT and ITT; however, it is accepted that there may be variability between job‐roles. Conversely, greater scores achieved by trained soldiers on the seated medicine ball throw and broad jump, who also have greater body mass than trainees, did not translate to faster times for the casualty drag as would be expected. This result is in contrast to research demonstrating that one‐person drags are performed faster by those individuals who are taller and heavier (Canino et al., 2019; von Heimburg et al., 2006; Reilly et al., 2013). Consequently, the outcome of faster casualty drag performance in trainees compared to trained soldiers may be explained by differences in intrinsic factors (e.g. motivation, test novelty, degree of functional training and competitiveness when performing the drag under “test” conditions) between the two groups. However, if muscular strength and endurance are equally important in the development of military performance and readiness (Kraemer et al., 2012; Kyröläinen et al., 2018) as well as key to soldiers physical performance, especially during operations (Mala et al., 2015; Pihlainen et al., 2020), emphasis should be given to strength and functional training during BT, ITT and through career.

Physical employment standards are designed with the principal aim of ensuring that the physical capabilities of individuals match that of their specific role (Rayson, 2000; Reilly et al., 2015). The trainees in this study were able to complete the representative military tasks to an acceptable standard suggesting that BT provides a sufficient training stimulus to prepare for their future job roles. In fact, the largest disparity between trainees and trained soldiers was in those tests and representative military tasks which are principally aerobically demanding tasks (i.e. 2 km run and loaded carriage); however, the performance on these tests still typically met or exceeded the minimum standards required for employment. Again, based on the literature, this is unsurprising, given that basic training has been demonstrated to substantially develop aerobic fitness (Dyrstad et al., 2006; Harwood et al., 1999; Rosendal et al., 2003; Santtila et al., 2008; Varley‐Campbell et al., 2018; Williams, 2005); even with a regression to the biological mean in aerobic performance (e.g. 2 km run and loaded carriage) demonstrated within BT (Burley et al., 2018; Rue et al., 2023).

The research is subject to a number of notable limitations. Firstly, participants involved in the study were not matched for role group (e.g. Royal Artillery, Royal Engineers and Army Air Corps) and since the later defined standards are allocated by role group, future research should seek to match data based on role‐related standards. Secondly, despite efforts to familiarise participants with each of the tests prior to measuring performance, trained soldiers would have had greater exposure to performing the role‐related movements and greater functional fitness as a result of their job‐role and could result in a regression to the mean (e.g. repeated measures artefact). Consequently, this may have contributed to some of the differences between the two groups. Future investigations should seek to provide a more substantial familiarisation period for all individuals prior to the collation of data to eliminate potential learning effects. Finally, data collection was ongoing as the COVID‐19 pandemic was approached, thus data collection was suspended for the recruit cohort resulting in a disparity of participant numbers between the two groups. A greater number of participant data for this group would have strengthened the dataset; however on this occasion, this was an unavoidable result of the pandemic.

## 5 CONCLUSION

The present study has shown that serving soldiers compared with trainees were older, heavier and scored higher on gym‐based strength and power tasks. Whereas, trainees performed better on the 2 km run, loaded carriage, tactical movement and casualty drag than trained soldiers. These results suggest that muscle strength and power further develop after BT and ITT and aerobic and anaerobic endurance decline. These changes maybe in due, in part, to ageing and/or reflect physiological adaptation of physical capabilities to meet the requirements of personnel performing their job‐tasks. These results support the notion that physical training for military job roles should be performed through career to ensure that all components of fitness are trained in addition to providing functional training to further improve role‐related task performance.

## CONFLICT OF INTEREST STATEMENT

The authors declare that they have no conflicts of interest with respect to this research.

## Supporting information

Table S1

Table S2

## Data Availability

Due to the sensitivity, data are not available.
