# Supplementary material for: A comparison of role‐related physical fitness between British Army trainees and trained soldiers
Source: Eur J Sport Sci. 2024 Dec 2;25(1):e12227. doi: 10.1002/ejsc.12227 (PMC11680188; doi:10.1002/ejsc.12227)
Supplement: Supplementary file 1 — Table S1 [file EJSC-25-e12227-s002.docx]

Table A.1: Hex Bar Deadlift: Estimated 1 repetition maximum (adapted from British Army Fitness test policy6).

| **1 Rep** | **40 kg** | **50 kg** | **60 kg** | **70 kg** | **80 kg** | **90 kg** | **100 kg** | **110 kg** | **120 kg** | **130 kg** | **140 kg** | **150 kg** |
| --- | --- | --- | --- | --- | --- | --- | --- | --- | --- | --- | --- | --- |
| **2 Reps** | 42 | 51 | 63 | 72 | 84 | 93 | 106 | 112 | 128 | 136 | 146 | 154 |
| **3 Reps** | 44 | 54 | 65 | 74 | 88 | 95 | 108 | 116 | 130 | 140 | 150 | 160 |
| **4 Reps** | 45 | 55 | 67 | 76 | 90 | 100 | 110 | 122 | 132 | 144 | 154 | 164 |
| **5 Reps** | 47 | 57 | 69 | 80 | 92 | 102 | 116 | 126 | 138 | 148 | 160 | 170 |
| **6 Reps** | 48 | 59 | 71 | 82 | 96 | 105 | 118 | 128 | 140 | 152 | 164 | 174 |
| **7 Reps** | 49 | 60 | 72 | 84 | 98 | 108 | 120 | 132 | 144 | 156 | 170 | 182 |
| **8 Reps** | 50 | 62 | 75 | 87 | 100 | 112 | 124 | 138 | 150 | 162 | 174 | 188 |
| **9 Reps** | 51 | 64 | 78 | 90 | 104 | 116 | 130 | 142 | 154 | 170 | 180 | 196 |
| **10 Reps** | 52 | 68 | 80 | 93 | 108 | 120 | 134 | 148 | 160 | 172 | 188 | 200 |
