# Supplementary material for: A comparison of role‐related physical fitness between British Army trainees and trained soldiers
Source: Eur J Sport Sci. 2024 Dec 2;25(1):e12227. doi: 10.1002/ejsc.12227 (PMC11680188; doi:10.1002/ejsc.12227)
Supplement: Supplementary file 2 — Table S2 [file EJSC-25-e12227-s001.docx]

Table A.2: Order of testing of the Representative Military Tasks (RMT), and weight of clothing and equipment.

| RMT | Clothing and Equipment |
| --- | --- |
| Loaded Carriage | Stage 1: Webbing (7.5 kg), Weapon (4.5 kg), Bergen (23-27 kg)  Stage 2: Webbing (7.5 kg), Weapon (4.5 kg), Daysack (8.5-12.5 kg) |
| Tactical Movement, Casualty Drag, Stretcher Carry | Helmet (1.3 kg), Body Armour (5.6 kg), Webbing (7.5 kg), Weapon (4.5 kg) |
| Vertical Lift | Helmet (1.3 kg), Body Armour (5.6 kg), Weapon (4.5 kg) |
| Repeated Carry, Incremental Lift | Helmet (1.3 kg) |
